# Predictive model for ulcerative colitis therapeutic response using clinical indicators and peripheral blood T-cell subsets

**DOI:** 10.3389/fimmu.2026.1905745

**Published:** 2026-09-01

**Authors:** Tian Pu, Chunru Wang, Ranran Feng

**Affiliations:** Department of Gastroenterology, The First Affiliated Hospital of Zhengzhou University, Zhengzhou, Henan, China

**Keywords:** biomarkers, machine learning, peripheral blood T-cell subsets, treatment response prediction, ulcerative colitis

## Abstract

**Objective:**

This study aimed to integrate clinical indicators with peripheral blood T-cell subsets to develop a prediction model for ulcerative colitis (UC), and to facilitate precise subtyping and individualized treatment decisions.

**Methods:**

A retrospective cohort of 346 UC patients (June 2023–June 2025) was randomly assigned to training (n=242) and validation (n=104) sets at a 7:3 ratio. Univariate analysis, Least absolute shrinkage and selection operator (LASSO) regression and multivariate logistic regression to identify independent influencing factors. Machine learning models—random forest (RF), gradient boosting, and logistic regression—were constructed using the selected core variables. Model discrimination, calibration, and clinical utility were assessed using the area under the receiver operating characteristic curve (AUC), calibration curves, and decision curve analysis (DCA).

**Results:**

According to week-14 response criteria, the training set comprised 158 responders (65.3%) and 84 non-responders (34.7%). Six indicators significantly associated with treatment response: albumin, C-reactive protein (CRP), tumor necrosis factor-alpha (TNF-α), interleukin-6 (IL-6), CD3+CD4+CD25+/CD3+CD4+ T-lymphocyte ratio, and CD4/CD8 ratio were as independent influencing factors. Among the models, RF exhibited the highest numerical AUC, with an AUC of 0.852 in the training set and 0.804 in the validation set. The calibration curve demonstrated good agreement between predicted and actual risks. DCA indicated higher net clinical benefit within a risk threshold range of 0.1–0.8.

**Conclusion:**

A predictive model for UC treatment response based on clinical indicators and peripheral blood T-cell subsets was constructed and validated, and may serves as an exploratory tool for individualized treatment decisions in UC.

## Introduction

Ulcerative colitis (UC) is a chronic, relapsing inflammatory bowel disease with an increasing prevalence that significantly impairs patients’ quality of life (1). Current standard treatment regimens primarily include aminosalicylates, corticosteroids, and biologic agents (2). However, approximately 30%–40% of patients exhibit primary non-response or loss of response to first-line biologics, and this uncertainty in treatment efficacy severely hampers precision therapy (3). At present, efficacy assessment is mostly based on post-treatment retrospective analyses, and effective tools for pre-treatment prediction of individual responses are lacking (4). The pathogenesis of UC involves complex interactions among genetic factors, gut microbiota, intestinal barrier function, and the immune system (5). Recently, the role of T cell-mediated immune dysregulation in determining therapeutic outcomes in UC has gained increasing attention. Patients with UC commonly present with an abnormal CD4/CD8 ratio, insufficient function of CD3+CD4+CD25+/CD3+CD4+ T cells, and excessive activation of effector T cells; these abnormalities are closely associated with disease activity and treatment resistance (6). A decline in CD3+CD4+ CD25+/CD3 +CD4+ T cell function may lead to insufficient immunosuppression, whereas activated CD4+ T cells exacerbate inflammation by promoting the release of pro-inflammatory cytokines such as tumor necrosis factor-α (TNF-α) and interleukin-6 (IL-6) (7). Clinical studies have demonstrated that a decreased baseline CD4/CD8 ratio, a reduced CD3+CD4+CD25+/CD3+CD4+ T cell ratio, and an elevated proportion of activated helper T cells are significantly correlated with biologic treatment failure (8). Furthermore, traditional biomarkers including C-reactive protein (CRP), albumin, TNF-α, and IL-6 are also important indicators for evaluating disease activity and predicting treatment response in UC. Nevertheless, existing studies have mostly focused on single indicators, and comprehensive predictive models integrating multidimensional clinical parameters, T cell immune status, and serum cytokines remain lacking. Machine learning techniques offer unique advantages in handling complex data and constructing predictive models. Consequently, this study aims to integrate baseline clinical characteristics (albumin, CRP), serum cytokines (TNF-α, IL-6), and peripheral blood T cell immune parameters (CD4/CD8 ratio, absolute CD4+ T cell count, percentage of activated helper T cells, and CD3+CD4+CD25+/CD3+CD4+ T cell ratio) from UC patients to build and validate a predictive model for treatment response, thereby providing a quantitative basis for personalized therapy in clinical practice.

## Patients and methods

### Study population

A total of 346 patients with UC who attended the Department of Gastroenterology and Inflammatory Bowel Disease Center of our hospital between June 2023 and June 2025 and completed 14 weeks of standardized biologic therapy were retrospectively enrolled. Sample size calculation was performed prior to the study. Based on the expected incidence of the primary outcome (treatment non-response) set at 35%–40% (derived from our center’s preliminary data and published reports (9)), a protocol aligned with the core statistical methods (multivariable logistic regression and machine learning models) was adopted. The calculation was performed using PASS 2021 software and validated with the “pwr” package in R 4.2.3. A two-sided significance level (α) of 0.05, a power (1-β) of 80%, and a 10% dropout rate were assumed, yielding a minimum required sample size of 280 cases. The actual enrolled sample (n=346) exceeded this requirement, and the statistical power was verified to be >85%, satisfying the needs of subsequent multivariable analysis and machine learning modeling. Inclusion criteria were as follows: (1) age ≥18 years; (2) diagnosis of UC according to the Consensus on Diagnosis and Treatment of Inflammatory Bowel Disease, confirmed by endoscopic and histopathological examination, with moderate-to-severe active disease (modified Mayo score ≥6); (3) first receipt of anti-TNF-α biologic therapy (infliximab or adalimumab) with completion of the 14-week induction phase; (4) complete baseline assessments performed before treatment, including clinical characteristics, laboratory parameters, endoscopic scores, peripheral blood T cell immune parameters (CD4/CD8 ratio, absolute CD4+ T cell count, percentage of activated helper T cells, and CD3+CD4+CD25+/CD3+CD4+ T cell ratio), and serum cytokines (TNF-α, IL-6); and (5) availability of complete clinical and endoscopic evaluation data after treatment for efficacy determination. Exclusion criteria were as follows: (1) coexisting infectious enteritis, intestinal tuberculosis, or other organic intestinal diseases; (2) severe cardiac, hepatic, or renal insufficiency, or malignancy; (3) active infection (e.g., tuberculosis, hepatitis B, hepatitis C) or immunodeficiency disorders; (4) pregnancy or lactation; (5) previous biologic therapy or intestinal surgery; (6) discontinuation of anti-TNF-α therapy during the 14-week induction phase due to non-medical reasons (e.g., loss of confidence, personal preference, poor adherence); (7) incomplete baseline or 14-week follow-up data that precluded efficacy group assignment.

### Data collection

Multidimensional information was systematically collected from the hospital’s electronic medical record system, the gastroenterology clinical database, and the dedicated follow-up system for research purposes to construct the predictive model. Baseline demographic and clinical characteristics were collected, including: (1) demographic data: age, sex, height, weight, body mass index (BMI); (2) disease characteristics: disease duration (months), disease extent (E1–E3), disease activity, and prior medication history (aminosalicylates, corticosteroids, immunosuppressants); and (3) lifestyle factors: smoking history (pack-years), alcohol consumption (g/day). Pre-treatment baseline assessment parameters were also collected, covering: (1) clinical activity indices: modified Mayo score and partial Mayo score; (2) routine laboratory parameters: albumin, C-reactive protein (CRP), erythrocyte sedimentation rate, platelet count; (3) endoscopic score: Mayo endoscopic score; (4) serum cytokines: interleukin-6 (IL-6), tumor necrosis factor-α (TNF-α), and interleukin-17 (IL-17) measured by flow cytometry or enzyme-linked immunosorbent assay; and (5) peripheral blood T cell immune parameters: CD4/CD8 ratio, absolute helper/inducer T lymphocyte count (absolute CD4+ T cell count), percentage of activated helper T cells, and CD3+CD4+CD25+/CD3+CD4+ T cell ratio measured by flow cytometry. Finally, details of the treatment regimen, which uniformly consisted of first-line anti-TNF-α therapy (infliximab or adalimumab), and efficacy outcomes at week 14 post-treatment were recorded. The primary outcome was the efficacy group assignment based on the clinical response criteria at week 14 (responder vs. non-responder). Secondary outcomes, which served to further characterize the responder group, included the clinical remission rate and the steroid-free clinical remission rate. All data were uniformly managed through an electronic data capture system and were collected by trained researchers following standardized procedures to ensure data completeness and accuracy.

### Outcome definitions

According to the ECCO-ESGAR-ESP-IBUS guidelines (10) and the UC-RESPONSE study protocol (11) for assessing response to biologic therapy, and considering the 14-week induction phase of this study, patients were divided into two groups. The responder group was defined as meeting any of the following criteria after 14 weeks of treatment: (1) clinical response: a decrease in partial Mayo score from baseline of ≥3 points and ≥30%, accompanied by a decrease in the rectal bleeding subscore from baseline of ≥1 point or achievement of 0-1 point; (2) treatment completion: no premature discontinuation of anti-TNF-α therapy due to drug-related adverse events or documented disease progression; and (3) adherence: biologic infusion adherence of no less than 85%. The supplementary criteria of treatment completion and infusion adherence are not independent confounding factors; premature therapy withdrawal driven by disease deterioration or drug adverse events essentially represents treatment failure, and insufficient adherence will distort objective clinical activity evaluation based on partial Mayo score, which jointly reflect comprehensive therapeutic response status of patients. The non-responder group was defined as meeting any of the following criteria after 14 weeks of treatment: (1) failure to achieve the above clinical response criteria; (2) symptom worsening: no improvement or an increase in the partial Mayo score from baseline; or (3) treatment discontinuation: premature withdrawal from treatment due to drug-related adverse events or documented disease progression without achieving clinical response at the time of withdrawal. All assessments of clinical activity indices and laboratory tests were performed by uniformly trained gastroenterologists using standardized evaluation procedures at two time points: before treatment initiation and after 14 weeks of treatment. Efficacy determination was performed independently by two researchers who were fully blinded to patients’ baseline clinical data, laboratory parameters and raw treatment allocation information. The inter-rater Cohen’s Kappa coefficient for outcome grouping reached 0.927, showing excellent judgment consistency between two assessors. In the event of disagreement in group assignment, arbitration was conducted by a third senior researcher from the study team according to predefined criteria. All clinical data and evaluation results were entered and managed using an electronic data capture system to ensure data accuracy, completeness, and traceability.

### Statistical analysis

Data analyses were performed using SPSS 26.0, R 4.2.3, and Python 3.8.5. Normally distributed continuous variables were expressed as mean ± standard deviation ( $\bar{x}$ ± s), and comparisons between groups were conducted using the independent-samples t-test. Categorical variables were expressed as number (percentage) [n(%)], and group comparisons were performed using the χ² test or Fisher’s exact test. Sample size calculation followed the guidelines for prediction model studies, with a two-sided significance level (α) of 0.05 and a power (1-β) of 80%. In addition, the “events per variable” (EPV) principle (at least 5–10 events per variable) was adopted to ensure model stability. With 6 independent predictors and 84 non-response events in the training set, the EPV reached approximately 14, which fully met the recommended EPV ≥10 threshold for clinical prediction models. No missing data were present for any of the core predictor variables (albumin, CRP, IL-6, TNF-α, CD3+CD4+CD25+/CD3+CD4+ T cell ratio, CD4/CD8 ratio) or the outcome variable (treatment response at week 14), as the inclusion criteria required complete baseline assessments and 14-week follow-up evaluations for all enrolled patients. Therefore, no imputation methods were applied in this study. The data set was randomly divided into a training set and a validation set at a ratio of 7:3 using stratified sampling implemented with the “caret” package in R 4.2.3 to ensure balanced distribution of outcome events between the two sets. In the training set, univariable analysis was first performed to screen for variables with P<0.05 using raw laboratory values. This initial screening step served to exclude variables with no marginal association with the outcome, thereby reducing the risk of overfitting and improving model parsimony while preserving clinically relevant candidates. All candidate continuous predictors were then processed with Z-score standardization to eliminate dimensional differences before variable shrinkage using LASSO regression, consistent with the scale-sensitive characteristic of penalized regression. Although entering all clinically relevant candidate variables directly into LASSO represents an alternative approach, we adopted this two-stage strategy to prioritize clinical interpretability and model stability, particularly given the limited number of events. For multivariate logistic regression and nomogram construction, standardized variables were converted back to original clinical measurement units for clinical interpretability. Finally, multivariable logistic regression was applied to identify independent influencing factors. Based on the results of multivariable analysis, machine learning models including logistic regression, random forest, and gradient boosting were further constructed using Python 3.8.5 and the sklearn library. For the logistic regression model, we used the default L2 regularization with the ‘liblinear’ solver, and the regularization parameter C was tuned using grid search over {0.01, 0.1, 1, 10, 100} with 10-fold cross-validation. For the random forest model, hyperparameter tuning was performed using grid search over the following ranges: n_estimators = {100, 200, 300}, max_depth = {5, 10, 15, 20}, min_samples_split = {2, 5, 10}, and min_samples_leaf = {1, 2, 4}. The optimal combination was n_estimators=200, max_depth=10, min_samples_split=5, and min_samples_leaf=2. For the gradient boosting machine, we tuned learning_rate = {0.01, 0.05, 0.1}, n_estimators = {100, 200}, max_depth = {3, 5, 7}, and subsample = {0.8, 1.0}, with the optimal parameters being learning_rate=0.05, n_estimators=200, max_depth=5, and subsample=0.8. All tuning procedures were performed exclusively on the training set using 10-fold cross-validation to prevent data leakage, and the final models were evaluated on the held-out validation set. Multiple strategies were integrated to mitigate model overfitting: stratified random data splitting balanced outcome distribution between training and validation sets; LASSO regression with 10-fold cross-validation shrank redundant variable coefficients to reduce model complexity; 1000-resample bootstrap internal validation was implemented for nomogram and logistic regression models; out-of-sample validation set was used to assess generalization performance of all machine learning algorithms; hyperparameter constraints on maximum tree depth and minimum leaf sample size were applied for random forest and gradient boosting machine to avoid excessive fitting to training noise. Receiver operating characteristic (ROC) curves were plotted using GraphPad Prism 9.0, and the area under the curve (AUC) was calculated to evaluate the predictive performance of the models. Furthermore, a nomogram prediction model was constructed using the “rms” package in R software, and internal validation was performed using the bootstrap method (1, 000 resamples). Calibration curves were plotted to assess calibration. In addition, SHAP values were calculated using the “shap” library in Python to evaluate model interpretability from both global (feature importance ranking and direction of contribution) and local (decomposition of risk contribution for individual patients) perspectives. Combined with the visualization function of the nomogram, this approach enabled clinical interpretability of the model’s predictions.

## Results

### Comparison of baseline characteristics between the training and validation sets

A total of 346 UC patients who completed the 14-week induction phase were enrolled in this study and randomly divided into a training set (n=242) and a validation set (n=104) at a ratio of 7:3. A flow diagram of patient selection is presented in **Supplementary Figure 1**. Of the 412 initially screened patients, 66 were excluded, and 346 eligible patients were enrolled in the final analysis. In the training set, based on the week-14 clinical response criteria, 158 patients (65.3%) were classified as responders and 84 (34.7%) as non-responders. In the validation set, 68 patients (65.4%) were responders and 36 (34.6%) were non-responders. Comparison of baseline data between the two sets showed no statistically significant differences in demographic characteristics (age, sex, BMI), disease characteristics (disease duration, Mayo endoscopic score), clinical laboratory parameters (albumin, platelet count, CRP, erythrocyte sedimentation rate), or serum cytokine and immunological markers (IL-6, IL-17, TNF-α, CD3+CD4+ CD25+/CD3+CD4+ T cell ratio, CD4/CD8 ratio, absolute lymphocyte count) (*P*>0.05). These results indicate that the data set partition was balanced, and the training and validation sets were well-matched (**Table 1**).

**Table 1:** Comparison of baseline characteristics between the training and validation sets.

Indicator		Training set (n=242)	Validation set (n=104)	*t/χ²*	*P*
Age (years)		38.42 ± 14.56	39.12 ± 15.23	0.404	0.686
Sex(n, %)	Male	142(58.68)	61(58.65)	0.052	0.819
	Female	100(41.32)	43(41.35)		
BMI(kg/m²)		22.16 ± 3.78	22.34 ± 3.65	0.410	0.682
Disease duration (months)		42.34 ± 38.56	41.67 ± 37.89	0.149	0.882
Albumin (g/L)		39.23 ± 5.34	39.45 ± 5.21	0.354	0.724
Platelet count (×10^9^/L)		312.56 ± 89.45	308.23 ± 87.34	0.416	0.678
CRP(mg/L)		18.45 ± 15.67	17.89 ± 14.98	0.309	0.758
ESR(mm/h)		26.78 ± 19.34	25.98 ± 18.67	0.356	0.722
Mayo endoscopic score		2.12 ± 0.78	2.08 ± 0.74	0.444	0.657
IL-6(pg/mL)		9.34 ± 7.23	8.98 ± 6.89	0.431	0.667
IL-17(pg/mL)		10.23 ± 6.78	9.87 ± 6.45	0.462	0.645
TNF-α(pg/mL)		8.45 ± 5.67	8.12 ± 5.34	0.505	0.614
Ratio of CD3+CD4+CD25+ to CD3+CD4+ T cells		8.37 ± 2.45	8.12 ± 2.38	0.491	0.624
CD4/CD8 ratio		2.38 ± 0.67	2.35 ± 0.65	0.382	0.703
Absolute lymphocyte count (cells/μL)		2483 ± 456	2456 ± 442	0.508	0.612
Absolute count of helper/inducer T lymphocytes (cells/μL)		1108 ± 234	1092 ± 228	0.588	0.557
Percentage of activated helper T cells (%)		15.17 ± 4.23	14.89 ± 4.12	0.569	0.572

### Univariate analysis of factors influencing treatment response in UC patients

Among the 242 patients in the training set, 158 (65.3%) were classified as responders and 84 (34.7%) as non-responders based on the clinical response criteria at week 14. Univariate analysis revealed statistically significant differences between the responder and non-responder groups in the training set for albumin, CRP, IL-6, TNF-α, the ratio of CD3+CD4+CD25+ to CD3+CD4+ T cells (Treg proportion), and the CD4/CD8 ratio (*P* < 0.05)(**Table 2**). Specifically, the responder group exhibited higher albumin levels, Treg proportion, and CD4/CD8 ratio, whereas the non-responder group showed elevated levels of CRP, IL-6, and TNF-α. No statistically significant differences were observed between the two groups for the remaining variables, including age, sex, BMI, disease duration, platelet count, erythrocyte sedimentation rate, Mayo endoscopic score, IL-17, absolute lymphocyte count, absolute helper/inducer T lymphocyte count, and percentage of activated helper T cells (*P*>0.05).

**Table 2:** Univariate analysis of factors influencing treatment response in UC patients.

Indicator		Responder group (n=158)	Non-responder group (n=84)	*t/χ²*	*P*
Age (years)		38.12 ± 14.23	38.98 ± 15.12	0.438	0.662
Sex	Male	93(58.9)	49(58.3)	0.003	0.954
	Female	65(41.1)	35(41.7)		
BMI(kg/m²)		22.23 ± 3.67	22.02 ± 3.98	0.411	0.681
Disease duration (months)		41.23 ± 37.45	44.34 ± 40.57	0.597	0.551
Albumin (g/L)		40.54 ± 5.10	36.79 ± 5.31	5.383	<0.001
Platelet count (×10^9^/L)		308.45 ± 87.34	320.23 ± 92.56	0.978	0.329
CRP(mg/L)		14.21 ± 12.36	26.32 ± 18.55	6.059	<0.001
ESR(mm/h)		25.89 ± 18.34	28.45 ± 20.12	0.999	0.319
Mayo endoscopic score		2.08 ± 0.76	2.19 ± 0.81	1.048	0.296
IL-6(pg/mL)		7.49 ± 6.76	12.35 ± 8.14	4.940	<0.001
IL-17(pg/mL)		10.12 ± 6.56	10.45 ± 7.12	0.368	0.713
TNF-α(pg/mL)		7.22 ± 4.56	10.68 ± 5.87	4.893	<0.001
Ratio of CD3+CD4+CD25+ to CD3+CD4+ T cells		9.10 ± 2.34	6.89 ± 2.13	7.320	<0.001
CD4/CD8 ratio		2.56 ± 0.58	2.02 ± 0.62	6.787	<0.001
Absolute lymphocyte count (cells/μL)		2512 ± 445	2430 ± 478	1.342	0.181
Absolute count of helper/inducer T lymphocytes (cells/μL)		1105 ± 230	1090 ± 235	0.482	0.630
Percentage of activated helper T cells (%)		14.89 ± 4.12	15.23 ± 4.35	0.621	0.535

### Multivariate logistic regression analysis of factors influencing treatment response in UC patients

**Figure 1:**
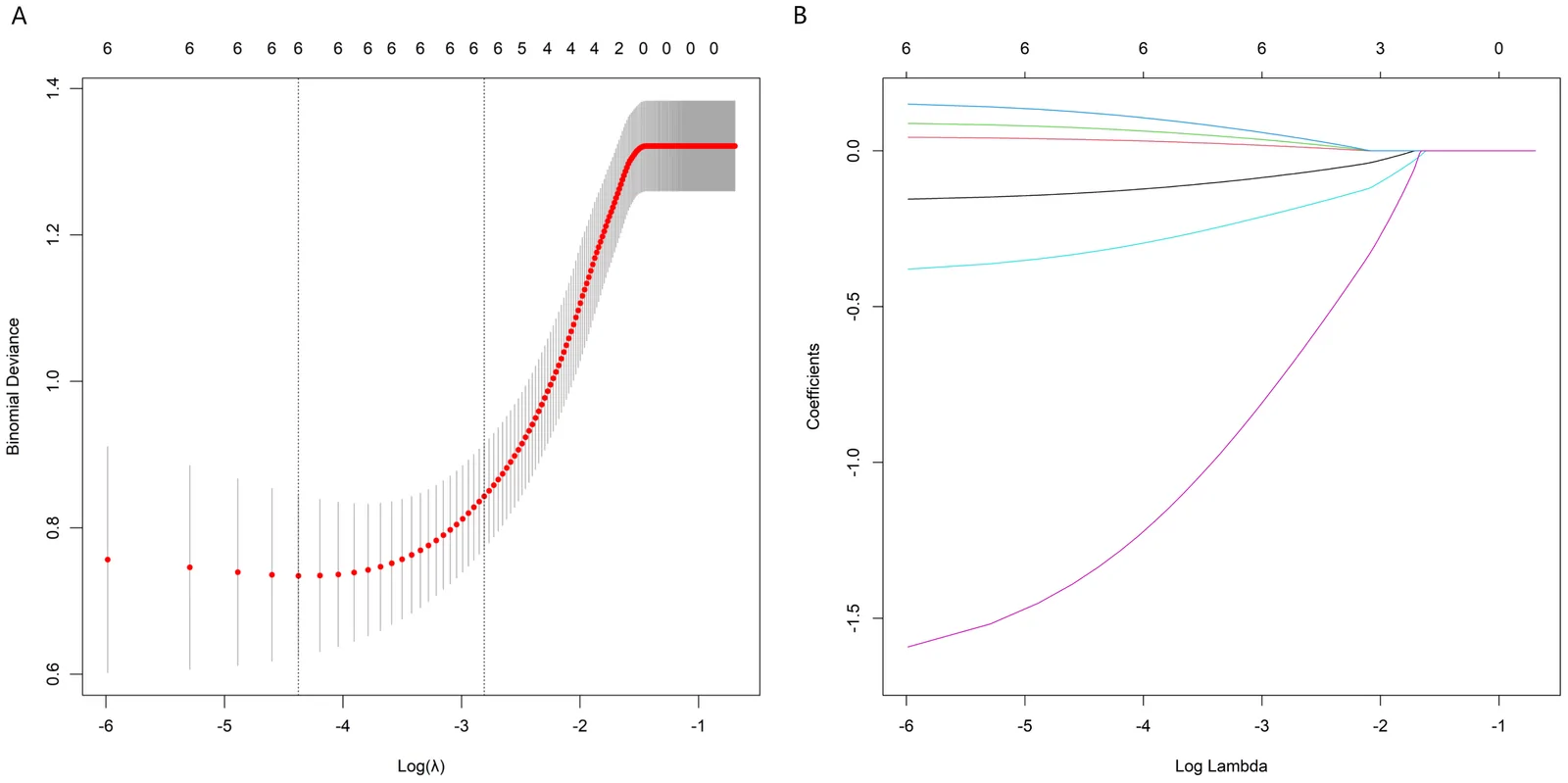
LASSO-penalized regression for candidate predictor screening. **(A)** Cross-validation binomial deviance curve. The x-axis represents log(λ), and the y-axis indicates binomial deviance. The vertical dashed lines denote λ_min_(left) and λ_1se_ (right). Numbers at the top of **(A)** represent the count of non-zero coefficients retained at corresponding λ values. **(B)** LASSO coefficient-path plot. The x-axis shows log(λ), and the y-axis displays regression coefficient values for each biomarker. Each colored line corresponds to one predictor variable. The numbers at the top of **(B)** indicate the number of predictors with non-zero coefficients at each λ. At the selected λ_1se_, all six candidate predictors maintained non-zero regression coefficients.

Using treatment response as the dependent variable (1 = non-responder group, 0 = responder group), all six indicators that achieved statistical significance in the univariate analysis (albumin, CRP, IL-6, TNF-α, CD3+CD4+CD25+/CD3+CD4+ T cell ratio, and CD4/CD8 ratio) were entered into the LASSO regression for variable selection (variable coding shown in **Supplementary Table 1**). The optimal variables were selected using 10-fold cross-validation with the λ-1se criterion (**Figure 1**). The λ_min_ and λ_1se_ values were 0.021 and 0.076, respectively, with corresponding cross-validation binomial deviances of 0.732 (SE = 0.041) and 0.748 (SE = 0.043). Notably, under the λ_1se_ criterion, none of the six biomarker coefficients were shrunk to zero, indicating that all predictors contained independent predictive information. Consequently, all six predictors were retained and included in the multivariate logistic regression model. The multivariate logistic regression analysis demonstrated that each of the six indicators was an independent predictor of non-response to treatment in UC patients (*P* < 0.05) (**Table 3**). Among these, albumin, the CD3+CD4+ CD25+/CD3+ CD4+ T cell ratio, and the CD4/CD8 ratio were protective factors, whereas CRP, IL-6, and TNF-α were risk factors.

**Table 3:** Multivariate logistic regression analysis of factors influencing treatment response in UC patients.

Indicator	β	SE	Wald	*P*	OR	95%CI
Albumin	-0.135	0.037	13.112	<0.001	0.874	0.812~0.941
CRP	0.054	0.014	14.877	<0.001	1.055	1.027~1.084
IL-6	0.096	0.027	12.642	<0.001	1.101	1.044~1.161
TNF-α	0.157	0.042	13.976	<0.001	1.170	1.078~1.270
CD3+CD4+CD25+/CD3+CD4+ T cell ratio	-0.422	0.091	21.503	<0.001	0.655	0.549~0.782
CD4/CD8 ratio	-0.510	0.147	12.036	<0.001	0.600	0.451~0.798

### Performance evaluation of machine learning models

**Figure 2:**
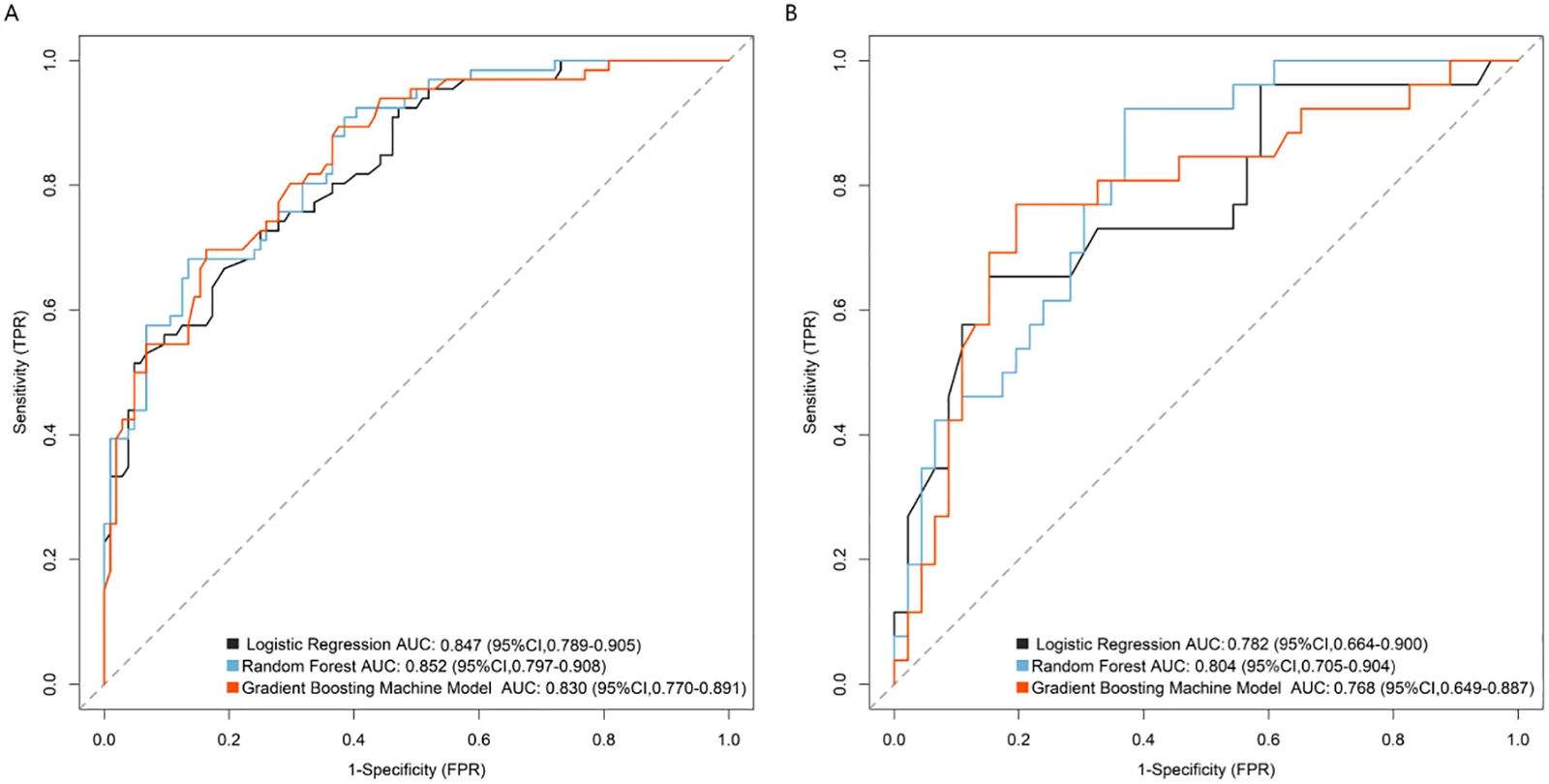
ROC curves of the prediction models in the training and validation cohorts. **(A)** ROC curves in the training set for logistic regression (AUC = 0.847), random forest (AUC = 0.852), and gradient boosting machine (AUC = 0.830). **(B)** ROC curves in the validation set for logistic regression (AUC = 0.782), random forest (AUC = 0.804), and gradient boosting machine (AUC = 0.768). The diagonal dashed line represents the reference line (AUC = 0.5).

Based on the six key predictors identified by univariate analysis (albumin, CRP, IL-6, TNF-α, CD3+CD4+CD25+/CD3+ T-cell ratio, and CD4/CD8 ratio), three machine learning predictive models—logistic regression, random forest, and gradient boosting machine—were further constructed to comprehensively evaluate and optimize their predictive performance for the target outcome. The random forest model achieved an AUC of 0.852 in the training set and 0.804 in the validation set, higher than logistic regression (training AUC 0.847, validation AUC 0.782) and gradient boosting machine (training AUC 0.830, validation AUC 0.768). Pairwise DeLong test for AUC comparison showed that random forest had significantly stronger discrimination than gradient boosting machine in the training set (P = 0.039), with marginal significance in the validation set (P = 0.047); no significant AUC difference was detected between random forest and logistic regression in either cohort (training set P = 0.261, validation set P = 0.183). The ROC curves in **Figures 2A, B** visually reflect the above discrimination differences among the three algorithms.

**Figure 3:**
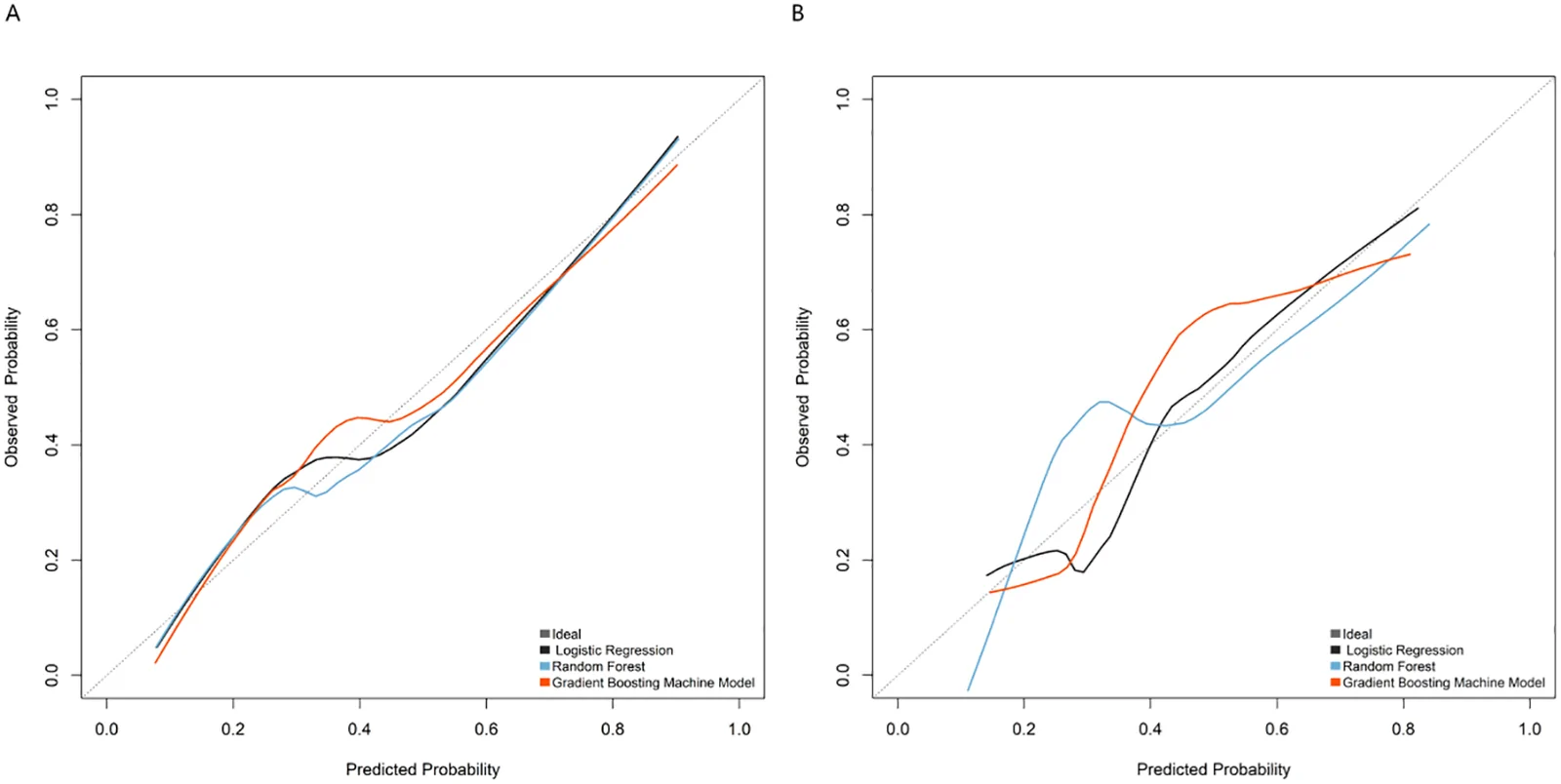
Calibration curves of the prediction models in the training and validation cohorts. **(A)** Calibration curves in the training set for logistic regression, random forest, and gradient boosting machine. **(B)** Calibration curves in the validation set for the three models. The diagonal solid line represents perfect calibration (predicted probability = observed probability). The closer the calibration curve to the diagonal line, the better the calibration performance. Brier scores and integrated calibration errors are reported in the main text.

**Figure 4:**
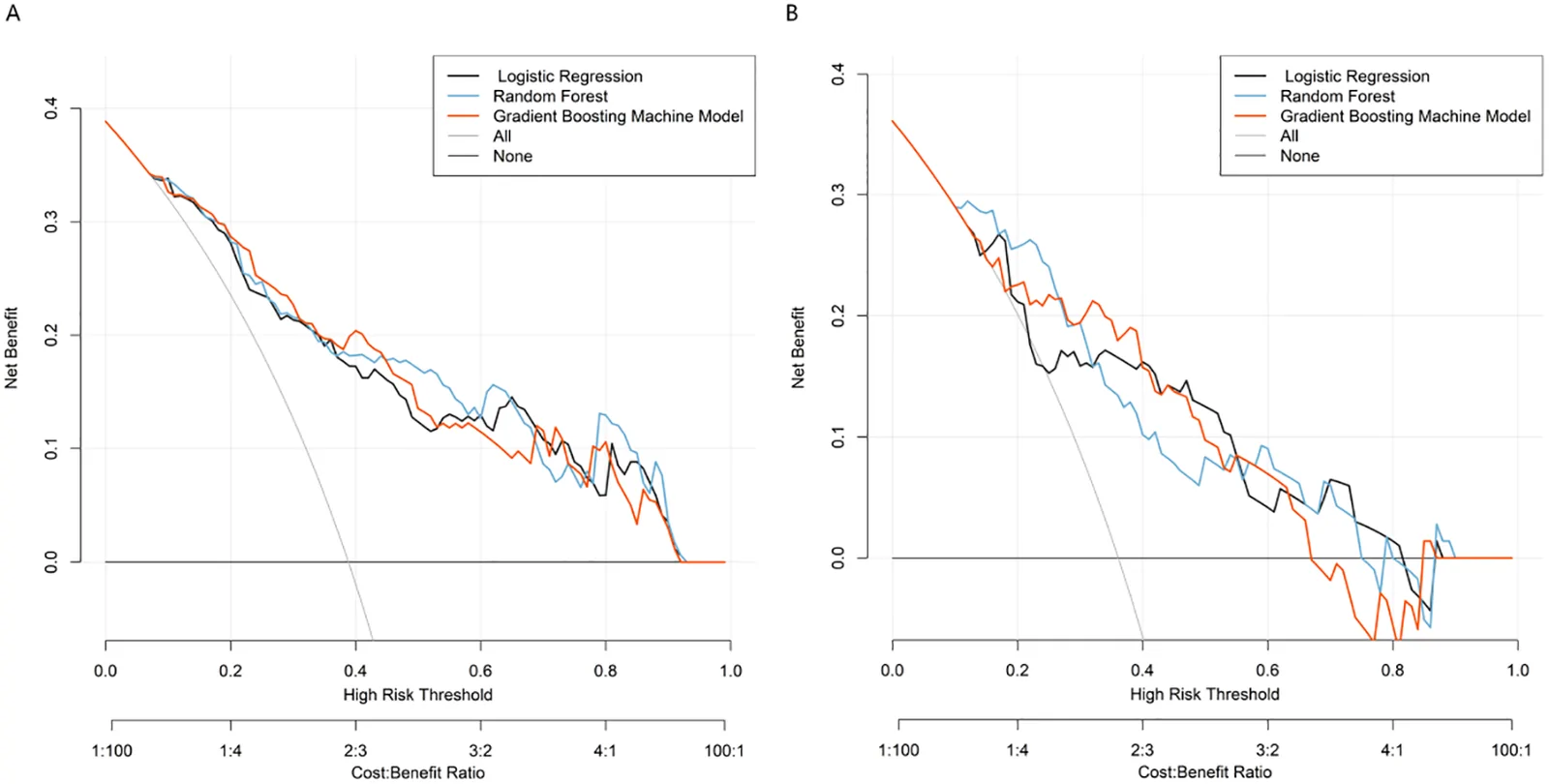
DCA of the prediction models in the training and validation cohorts. **(A)** DCA curves in the training set for logistic regression, random forest, and gradient boosting machine, with the “treat all” and “treat none” strategies shown as reference. **(B)** DCA curves in the validation set for the three models. The y-axis represents the net clinical benefit, and the x-axis represents the threshold probability. A model with higher net benefit across a wider range of threshold probabilities is considered clinically more useful.

Random forest yielded a training-set Brier score of 0.112 and integrated calibration error of 0.021, with corresponding validation-set values of 0.131 and 0.028; these metrics were lower than those of logistic regression (training: 0.135, 0.036; validation: 0.159, 0.047) and gradient boosting machine (training: 0.148, 0.042; validation: 0.172, 0.053). Consistently, calibration curves in **Figures 3A, B** illustrated that random forest’s predicted risks closely matched the actual observed risks, with only minor deviation at high probability levels in the validation cohort, which verified reliable calibration capacity. In addition, decision curve analysis (**Figures 4A, B**) demonstrated that across a wide threshold probability range of 0.1–0.8, the clinical net benefit of this model was consistently superior to the extreme strategies of ‘treat all’ or ‘treat none, ‘ with optimal net benefit within the clinically common threshold range (0.1–0.5). Consequently, the random forest model was selected as the preferred predictive model for this study after comprehensive evaluation of discrimination, calibration, prediction error and clinical net benefit.

### Interpretability assessment of model predictions

**Figure 5:**
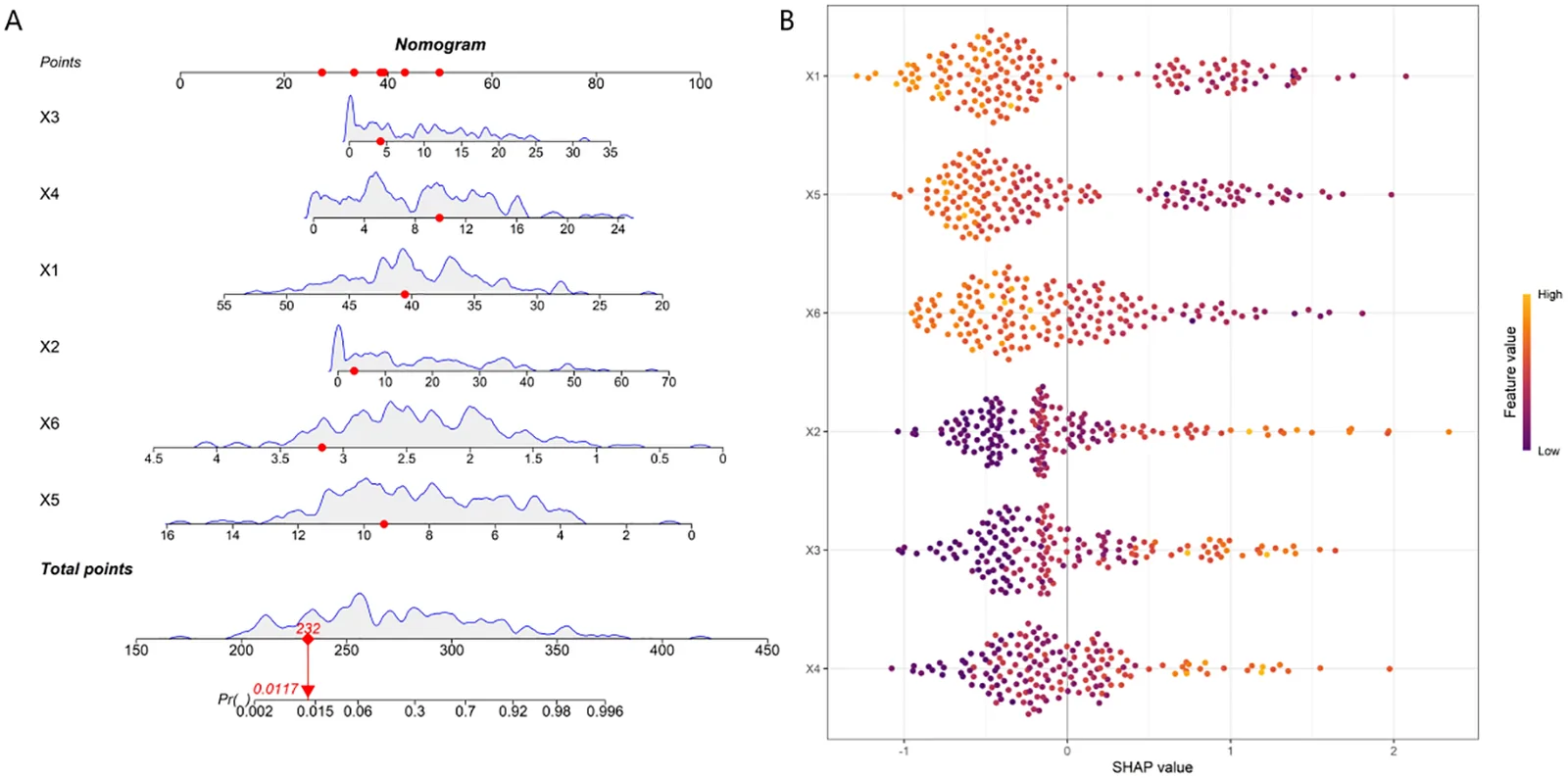
Model interpretability analysis. **(A)** Nomogram for predicting the risk of treatment non-response in ulcerative colitis patients. To use the nomogram, locate each predictor on its corresponding axis, draw a vertical line upward to the “Points” scale to obtain the score for each predictor, sum all scores to obtain the total points, and draw a vertical line from the “Total Points” axis downward to the “Risk of Non-response” axis to read the predicted probability. **(B)** SHAP summary plot showing the feature importance ranking and the direction of contribution for each predictor. The x-axis represents the SHAP value (impact on model output), and the y-axis lists the predictors in descending order of importance. Red indicates higher predictor values, and blue indicates lower predictor values. Positive SHAP values indicate increased risk of non-response, while negative SHAP values indicate decreased risk. X1: albumin; X2: CRP; X3: IL-6; X4: TNF-α; X5: CD3+CD4+CD25+/CD3+CD4+ T cell ratio; X6: CD4/CD8 ratio.

Using the six core predictors identified by univariate analysis (albumin, CRP, IL-6, TNF-α, CD3+CD4+CD25+/CD3+CD4+ T cell ratio, and CD4/CD8 ratio), a nomogram model (**Figure 5A**) was constructed based on the multivariate logistic regression model to visualize the prediction of treatment non-response. Meanwhile, SHAP analysis was performed on the random forest model to quantify the predictive contribution and direction of each feature (**Figure 5B**). SHAP analysis further clarified the relative importance ranking of the features: from highest to lowest impact, X2 (CRP), X5 (CD3+CD4+CD25+/CD3+CD4+ T cell ratio), X6 (CD4/CD8 ratio), X1 (albumin), X4 (TNF-α), and X3 (IL-6). Among these, X2 (CRP), X4 (TNF-α), and X3 (IL-6) showed positive SHAP values (risk-increasing direction), whereas X1 (albumin), X5 (CD3+CD4+CD25+/CD3+CD4+ T cell ratio), and X6 (CD4/CD8 ratio) showed negative SHAP values (risk-decreasing direction). To use the nomogram in clinical practice, clinicians should follow these steps: (1) locate each of the six predictors (albumin, CRP, IL-6, TNF-α, CD3+CD4+CD25+/CD3+CD4+ T cell ratio, and CD4/CD8 ratio) on the corresponding axis; (2) draw a vertical line upward to the ‘Points’ scale to obtain the score for each predictor; (3) sum all individual scores to obtain the total points; (4) locate the total points on the ‘Total Points’ axis and draw a vertical line downward to the ‘Risk of Non-response’ axis to read the predicted probability of treatment non-response. The example patient shown in **Figure 5A** illustrates this process, with a total score corresponding to an approximately 65% predicted risk of non-response.

## Discussion

UC is a chronic, relapsing, non-specific inflammatory bowel disease. The treatment response to UC exhibits substantial heterogeneity, with approximately 30%–40% of patients showing primary non-response or secondary loss of response to first-line biologic agents. Currently, there is a lack of effective clinical tools to accurately predict individual treatment outcomes prior to therapy (12, 13). In this study, we innovatively integrated clinical indicators (albumin, CRP) with peripheral blood T-cell immune parameters and serum cytokines (IL-6, TNF-α, CD3+CD4+CD25+/CD3+CD4+ T-cell ratio, and CD4/CD8 ratio) in a multidimensional manner. Consequently, we developed and validated a random forest model for personalized prediction of treatment response in UC patients. This model demonstrated stable and reliable predictive performance in both the training and validation cohorts (with AUCs of 0.852 and 0.804, respectively). Moreover, through LASSO regression, multivariable logistic regression, and comparisons among multiple machine learning algorithms, we ensured the robustness of the predictive variables and the optimal performance of the model. More importantly, we performed an in-depth interpretability analysis of the model by combining a nomogram with SHAP analysis. This approach not only quantified the relative importance of each predictor but also clarified the risk relationship between each predictor and treatment non-response, thereby providing new insights and a basis for understanding the immune mechanisms underlying differential treatment efficacy and for achieving precise clinical intervention in UC.

The six core predictors identified in this study profoundly reflect the complex pathophysiological nature of UC, which involves adaptive immune dysregulation, systemic inflammatory response disturbance, intestinal barrier damage, and impaired nutritional status. As verified by SHAP analysis, CRP, the CD3+CD4+CD25+/CD3+CD4+ T-cell ratio, the CD4/CD8 ratio, albumin, TNF-α, and IL-6 were the key predictors, ranked in descending order of contribution. Among these, CRP and T-cell subset-related immune indicators showed particularly outstanding predictive value, with SHAP contribution values significantly higher than those of the other variables. This finding highlights the central roles of adaptive immune homeostasis and systemic inflammatory burden in regulating treatment response in UC (14, 15).

CRP, the highest-contributing core predictor, is a classic marker of systemic inflammation. Persistent mucosal inflammation in UC induces the release of numerous pro-inflammatory cytokines, which in turn stimulate hepatic CRP synthesis. The level of CRP directly reflects the degree of intestinal inflammatory infiltration, the extent of mucosal damage, and the systemic inflammatory burden (16). High CRP levels can further exacerbate intestinal epithelial injury and immune cell infiltration by activating the complement system and amplifying local inflammatory cascades, thereby perpetuating chronic inflammation. Consequently, elevated baseline CRP significantly increases the risk of non-response to UC therapy and serves as the primary indicator for assessing disease severity and treatment response potential (17).

The CD3+CD4+CD25+/CD3+CD4+ T-cell ratio and the CD4/CD8 ratio, ranked as the second and third most contributory protective immune indicators, directly reflect the level of adaptive immune tolerance and homeostasis in the body. Regulatory T cells (CD3+CD4+CD25+) can suppress excessive activation of pro-inflammatory T cells, such as Th1 and Th17 cells, by secreting anti-inflammatory cytokines like IL-10 and TGF-β, thereby maintaining intestinal mucosal immune tolerance. A higher ratio of these cells among CD4+ T cells indicates stronger intestinal immunosuppressive function, which favors inflammatory resolution. The CD4/CD8 ratio characterizes the balance of T-cell subsets; a decreased ratio suggests aberrant proliferation and activation of cytotoxic CD8+ T cells, which directly mediate intestinal epithelial cell apoptosis and exacerbate mucosal damage. Together, these two indicators determine the direction of the immune response and constitute the key immunological basis for regulating treatment response in UC (18).

Albumin, as a nutritional-gut barrier-inflammatory composite marker, is an important protective factor. In UC patients, ulceration and increased vascular permeability of the intestinal mucosa lead to substantial protein leakage. Concurrently, chronic inflammation consumes nutrients, and impaired intestinal absorption further induces hypoalbuminemia. Albumin not only maintains nutritional status but also participates in intestinal mucosal repair, antioxidant activities, and immune regulation. Reduced albumin levels directly compromise intestinal epithelial regeneration, disrupt the integrity of the physical gut barrier, promote bacterial translocation, and sustain inflammatory activation, ultimately diminishing the response to pharmacotherapy (19).

TNF-α and IL-6, as downstream key pro-inflammatory cytokines, are involved in amplifying local and systemic inflammatory responses. TNF-α directly induces intestinal epithelial cell apoptosis and disruption of tight junctions, initiating mucosal damage. IL-6 promotes Th17 cell differentiation and induces acute-phase protein synthesis, thereby maintaining chronic inflammation. Both cytokines mediate immune cell infiltration into the intestinal mucosa and an imbalance between pro-inflammatory and anti-inflammatory forces, aggravating intestinal pathological injury. They are important mediators of disease activity and treatment resistance in UC. However, compared with the overall inflammatory burden and immune homeostasis, the independent predictive contribution of a single pro-inflammatory cytokine is relatively limited, which is consistent with the SHAP analysis results of the present study (20).

The core innovation of this study lies in breaking through the limitations of traditional prediction models that mainly rely on clinical inflammatory indicators, achieving a multidimensional integration of clinical indicators, serum cytokines, and T-cell immune parameters. Our prediction model demonstrates that treatment outcomes in UC are not determined by a single factor, but rather by the combined effects of traditional inflammatory burden (CRP, albumin), T-cell immune status (CD3+CD4+CD25+/CD3+CD4+ T-cell ratio and CD4/CD8 ratio), and pro-inflammatory cytokine levels (TNF-α and IL-6). This finding perfectly is consistent with the theory of complex interactions between innate and adaptive immunity in the pathogenesis of UC (21) and provides a novel immunological perspective for understanding differential treatment responses.

Methodologically, this study combined the strengths of traditional statistical methods and modern machine learning techniques. We initially screened variables using univariate analysis, then performed feature compression via LASSO regression to prevent overfitting. Subsequently, we used multivariable logistic regression to determine the independent roles of each factor, and finally employed a random forest algorithm to construct a model with stronger predictive performance and non-linear fitting capability. This combined strategy ensured both statistical rigor and enhanced predictive accuracy. Comparison among three machine learning models showed that the random forest model performed best in both the training cohort (AUC = 0.852) and the validation cohort (AUC = 0.804), validating its advantage in handling high-dimensional data and capturing non-linear relationships (22). Although the AUC differences between random forest and logistic regression were numerically modest (0.852 vs. 0.847 in the training set, and 0.804 vs. 0.782 in the validation set), the random forest model yielded consistently better calibration (integrated calibration error: 0.021 vs. 0.036 in training; 0.028 vs. 0.047 in validation) and lower Brier scores (0.112 vs. 0.135 in training; 0.131 vs. 0.159 in validation). These differences, while not large, suggest that random forest provides more reliable probability estimates across the full risk spectrum, which is clinically relevant for individualized decision-making. Therefore, we selected random forest as the preferred model based on its combined advantages in discrimination, calibration, and predictive stability, rather than on AUC alone. Comparison with previously published prediction models for UC treatment response reveals both similarities and differences. A recent systematic review and meta-analysis of 30 studies involving 7, 452 UC patients reported a pooled AUC of 0.84 (95% CI: 0.77-0.92) for prognostic prediction models, with common predictors including age, C-reactive protein (CRP), albumin, hemoglobin, disease extent, and Mayo scores (23). For biologic therapy specifically, a nomogram incorporating mean mucosal eosinophil density and CRP for predicting vedolizumab response in 84 UC patients achieved an AUC of 0.867 (95% CI: 0.781–0.953) (24). A risk score for corticosteroid failure in acute UC, developed from 682 presentations, reported AUCs of 0.76 (95% CI: 0.69–0.83) and 0.78 (95% CI: 0.71–0.85) for partial and full models, respectively (25). However, most existing models rely predominantly on conventional inflammatory markers and lack immune cell profiling. Our model, which integrates T-cell subset parameters (CD3+CD4+CD25+/CD3+CD4+ T cell ratio and CD4/CD8 ratio) with clinical and inflammatory indicators, achieved higher AUCs (0.852 in training, 0.804 in validation) and provides novel mechanistic insights into the immune basis of treatment response. Additionally, the application of SHAP analysis in our study offers granular interpretability that is absent in most previous models, enabling clinicians to understand not only the prediction but also the contribution of each factor at the individual patient level. These features collectively distinguish our model from prior work and underscore its potential for clinical translation. In summary, the random forest predictive model developed in this study based on albumin, inflammatory factors, and T-cell subset-related immune indicators exhibits favorable predictive accuracy, calibration, and clinical applicability. These findings suggest that the model provides an objective and reliable quantitative basis for individualized diagnosis and treatment decisions, and holds potential for clinical translation pending further external validation.

Furthermore, the application of SHAP interpretability analysis is a major highlight of this study. It objectively quantified the contribution ranking of each feature and clearly demonstrated the direction of positive or negative contribution of each feature to individual predictions—CRP exhibited a positive risk factor contribution, whereas the CD3+CD4+CD25+/CD3+CD4+ T-cell ratio, the CD4/CD8 ratio, and albumin showed protective factor contributions. This “white-box” approach greatly enhances clinicians’ trust in and understanding of the model’s decision-making process, advancing predictive models from mere “predictive performance” toward “clinically trustworthy” tools. Together with the visualization function of the nomogram, this study may improve interpretability and support further clinical evaluation. Through the combined application of the nomogram and SHAP analysis, this study not only constructed a visualized individualized prediction tool but also elucidated the decision-making mechanism of the model from the perspective of feature importance, significantly enhancing the clinical interpretability and practicality of the prediction results, thereby providing a reliable theoretical basis and practical guidance for precise diagnosis and treatment decisions for patients. To further quantify the incremental clinical value of T-cell subset markers beyond conventional inflammatory indicators, we performed a *post-hoc* analysis comparing model performance with and without the inclusion of CD3+CD4+CD25+/CD3+CD4+ T cell ratio and CD4/CD8 ratio. When these two immune parameters were excluded, the random forest AUC decreased from 0.852 to 0.813 in the training set and from 0.804 to 0.769 in the validation set, indicating that T-cell subset markers provided an absolute AUC gain of approximately 0.039 and 0.035, respectively. This incremental improvement is clinically meaningful, particularly in the intermediate-risk range where prediction uncertainty is highest. Moreover, SHAP analysis ranked these immune parameters as the second and third most important predictors (after CRP), underscoring their substantial contribution beyond conventional markers. These findings suggest that incorporating T-cell subset analysis into routine prediction workflows could enhance risk stratification, especially for patients with borderline inflammatory marker levels where clinical decisions are most challenging.

This study has several limitations. First, this was a single-center retrospective study, and although multiple anti-overfitting strategies were applied and the dataset was randomly split into training and validation sets, the validation performed in this study constitutes internal validation rather than true external validation. Although internal validation was performed using the bootstrap method (1, 000 resampling iterations), the generalizability and external validity of the model still require further testing in multicenter, prospective external cohorts. Second, all predictive variables were derived from baseline assessments, and dynamic changes during treatment (e.g., the rate of CRP decrease at week 4 or week 8) were not incorporated. Future studies could explore the development of dynamic prediction models to further improve predictive accuracy. Third, this study only included patients receiving first-line anti-TNF-α therapy (infliximab or adalimumab). Whether the predictive model can be generalized to other biologic classes (e.g., anti-IL-12/23 or anti-integrin agents) requires further investigation. Fourth, detection of the Treg ratio and the CD4/CD8 ratio relies on flow cytometry, and the standardization and generalizability of this method require further validation. Regarding the clinical applicability of flow cytometry-based T-cell subset measurements, we acknowledge that this technology is not universally available in all gastroenterology centers, particularly in resource-limited settings. Flow cytometry requires specialized equipment, trained personnel, and quality control procedures, which may increase operational costs and limit widespread adoption. However, in tertiary referral centers and specialized inflammatory bowel disease units where biologic therapies are commonly administered, flow cytometry is increasingly available as part of routine immunological monitoring. The incremental predictive value of T-cell subset indicators (as demonstrated by SHAP analysis) may justify the additional cost, particularly for patients with high baseline inflammatory burden or prior treatment failure, where accurate pre-treatment risk stratification could inform more rational therapeutic decisions. Future studies should evaluate the cost-effectiveness of incorporating these immune biomarkers into routine prediction workflows to better define their value in different healthcare settings.

In summary, based on six indicators—albumin, CRP, IL-6, TNF-α, the CD3+CD4+CD25+/CD3+CD4+ T-cell ratio, and the CD4/CD8 ratio—this study successfully constructed and validated a machine learning prediction model for treatment response in UC. The model exhibited good predictive performance, with AUCs of 0.852 and 0.804 in the training and validation cohorts, respectively, as well as good calibration and clinical net benefit. SHAP analysis identified CRP and T-cell subsets as core influential variables, revealing the intrinsic logic by which systemic inflammation and immune homeostasis regulate treatment response. This exploratory model, pending external validation, may provide a preliminary reference for personalized UC therapy, but its clinical application should be interpreted cautiously until confirmed in independent external cohorts Future multicenter prospective studies with external validation and incorporation of dynamic indicators are needed to further optimize model performance.

## Data Availability

The raw data supporting the conclusions of this article will be made available by the authors, without undue reservation.

## Supplementary material

Supplementary Figure 1 Flow diagram of patient selection. A total of 412 UC patients receiving biologic therapy were initially screened. After applying inclusion and exclusion criteria, 66 patients were excluded, and 346 patients were enrolled in the final analysis. The cohort was randomly divided into a training set (n=242, 70%) and a validation set (n=104, 30%) at a 7:3 ratio. Responder and non-responder proportions at week 14 are shown for each set.
