# Supplementary material for: Predictive model for ulcerative colitis therapeutic response using clinical indicators and peripheral blood T-cell subsets
Source: Front Immunol. 2026 Sep 1;17:1905745. doi: 10.3389/fimmu.2026.1905745 (PMC13574797; doi:10.3389/fimmu.2026.1905745)
Supplement: Supplementary file 1 [file Table1.docx]

**Supplemental Table 1.** Variable coding

| Variable | Meaning | Assignment |
| --- | --- | --- |
| X1 | Albumin | Continuous variable |
| X2 | CRP | Continuous variable |
| X3 | IL-6 | Continuous variable |
| X4 | TNF-α | Continuous variable |
| X5 | CD3+CD4+CD25+/CD3+CD4+ T cell ratio | Continuous variable |
| X6 | CD4/CD8 ratio | Continuous variable |
| Y | Treatment response | Responder group =0, Non-responder group =1 |
